# Therapeutic Effects of Milk Thistle (*Silybum marianum* L.) and Artichoke (*Cynara scolymus* L.) on Nonalcoholic Fatty Liver Disease in Type 2 Diabetic Rats

**DOI:** 10.1155/2022/2868904

**Published:** 2022-02-11

**Authors:** Aida Doostkam, Mohammad Fathalipour, Mohammad Hossein Anbardar, Azar Purkhosrow, Hossein Mirkhani

**Affiliations:** ^1^Shiraz Nephro-Urology Research Center, Shiraz University of Medical Sciences, Shiraz, Iran; ^2^Department of Pharmacology and Toxicology, Faculty of Pharmacy, Hormozgan University of Medical Sciences, Bandar Abbas, Iran; ^3^Department of Pathology, Shiraz University of Medical Sciences, Shiraz, Iran; ^4^Department of Pharmacology, School of Medicine, Shiraz University of Medical Sciences, Shiraz, Iran; ^5^Medicinal and Natural Products Chemistry Research Center, Shiraz University of Medical Sciences, Shiraz, Iran

## Abstract

**Background:**

At present, nonalcoholic fatty liver disease (NAFLD) does not have an approved pharmacologic therapy. The present study investigated the protective effects and possible mechanisms of milk thistle (*Silybum marianum* L.) and artichoke (*Cynara scolymus* L.) in treating NAFLD in type 2 diabetic rats.

**Methods:**

The NAFLD was established in rats after four weeks of type 2 diabetes induction. The animals were treated with pharmaceutical preparations of milk thistle (Livergol®) and artichoke (Atheromod-B®) extracts for eight weeks. After the end of the intervention, oral glucose tolerance, the serum parameters of oxidative stress, liver functional tests, and lipid profiles were evaluated. Histopathological changes were assessed by hematoxylin and eosin staining.

**Results:**

Treatment with preparations of milk thistle and artichoke nonsignificantly improved glucose tolerance in diabetic rats. Both preparations significantly improved serum superoxide dismutase activity and the level of malondialdehyde. Although treatment with milk thistle reduced serum activity of aspartate aminotransferase and serum levels of triglyceride (TG), total cholesterol, and low-density lipoprotein-cholesterol, artichoke extracts only attenuated the serum level of TG. Milk thistle also effectively protected the liver from histological changes.

**Conclusions:**

Milk thistle could be a promising pharmacological option for the treatment of NAFLD. Nonetheless, long-term randomized clinical trials are necessary to confirm the observed results.

## 1. Introduction

Diabetes mellitus (DM) is a common metabolic disorder characterized by high plasma glucose over a long period due to defects in insulin synthesis, insulin receptor, or postreceptor signaling pathway events. Chronic hyperglycemia is the leading cause of microvascular and macrovascular complications of DM [1].

More than 90% of diabetic patients with diabetes are afflicted by type 2 diabetes mellitus (T2DM). In type 2 diabetes, elevated plasma glucose and free fatty acid levels lead to oxidative stress, *β*-cell dysfunction, insulin resistance, and metabolic syndrome [2, 3]. One of the manifestations of these cellular and molecular disorders is a nonalcoholic fatty liver disease (NAFLD), a chronic liver disease [4].

NAFLD is a pathological condition characterized by hepatic fat accumulation not caused by the consumption of alcohol. It consists of a spectrum of liver diseases, from simple steatosis to steatohepatitis, hepatic fibrosis, chronic cirrhosis, and hepatocellular carcinoma [5]. In addition to type 2 diabetes, obesity and hypertriglyceridemia are the main coexisting conditions associated with NAFLD [6].

Milk thistle (*Silybum marianum* L.) is originally a native plant of Southern Europe through Asia with the active compound silymarin, a natural flavonoid. Silymarin consists of silybin, silychristin, silydianin, and isosilybin [7], and it is widely used as a nonprescription agent in liver diseases [8]. Several studies have indicated its promising antifibrotic activity in experimental liver injury [8, 9]. Reports show that it decreases oxidative stress by increasing glutathione levels [10]. Silymarin may decrease hepatic inflammation by inhibiting the lipo-oxygenase cycle and reducing leukotrienes and Kupffer cell function in animals [11]. In Iran, silymarin is sold as tablets under the proprietary name of Livergol®.

Artichoke (*Cynara scolymus* L.) is a perennial plant native to the Mediterranean region. Anti-hyperlipidemic effects of artichoke extract have been documented in clinical and experimental studies [12, 13]. Further studies have shown the inhibitory effects of artichoke extract on HMG-CoA reductase [14] and phosphatidate phosphohydrolase [15] in the cholesterol and triglyceride biosynthesis pathways, respectively. Some reports show that the artichoke extract reduces oxidative stress by reducing reactive oxygen species production and lipid peroxidation [13, 16]. Several researchers have indicated that artichoke promotes insulin secretion, sensitivity, and plasma glucose reduction in animal models of diabetes [17, 18]. In addition to this effect, the administration of artichoke extract has had beneficial effects in preventing type 2 DM and NAFLD in experimental animals [19, 20]. It is believed that cynarin, the most crucial active compound of the artichoke extract, is responsible for the cholagogue and choleretic properties of the plant [21]. In Iran, artichoke is sold as tablets under the proprietary name of Atheromod-B®.

Some pharmacological agents improve insulin resistance and hyperlipidemia and increase liver antioxidant defenses, but none is approved for NAFLD [22, 23]. Although recent studies have shown the therapeutic effects of milk thistle and artichoke for certain pathological conditions, the impact of these plants on NAFLD treatment is still unknown. In Iran, these plants are widely used for their putative hepatoprotective effects as pharmaceutical products.

The present study aimed (1) to assess whether intervention with pharmaceutical preparations of milk thistle and artichoke extracts in streptozotocin-nicotinamide-induced diabetic rats would improve the plasma oxidative stress, hepatic marker parameters, biochemical markers, and glucose tolerance and (2) to investigate whether these two plants declined the lipid accumulation in the rat liver.

## 2. Materials and Methods

### 2.1. Materials

Livergol® (lot. number: 0443) and Atheromod-B® (lot. number: 740164) were purchased from Goldaru (Isfahan, Iran) and Barij Essence (Isfahan, Iran) Pharmaceutical Companies, respectively. Livergol® and Atheromod-B® contain the extracts of milk thistle and artichoke, respectively. Streptozotocin (STZ; cat. number: S0130) and nicotinamide (NA; cat. number: N0636) were obtained from Sigma-Aldrich Chemical Company (Steinheim, Germany).

### 2.2. Animals

Forty male Sprague–Dawley rats (230–250 g) were obtained from the Center of Comparative and Experimental Medicine (Shiraz, Iran). Animals were kept under standard lighting (12-hour light/dark cycles), humidity (35–45%), and temperature (24°C ± 2°C).

The rats were randomly allocated to four groups of ten rats each. They included nondiabetic normal rats (normal group), type 2 diabetic rats (diabetic group), type 2 diabetic rats that received milk thistle (M group), and type 2 diabetic rats that received artichoke (A group).

### 2.3. Induction of Type 2 Diabetes Mellitus

To induce type 2 diabetes mellitus, NA (110 mg/kg) was injected into animals 15 min before a single intraperitoneal administration of STZ (50 mg/kg). Seven days after induction, fasting blood sugar (FBS) levels were checked using a Glucometer (Accu-check®, Germany) and rats with FBS levels between 126 and 260 mg/dl were considered as type 2 diabetic animals [24]. The confirmation day was considered the first day of diabetes. Four weeks after the confirmation of diabetes, FBS was determined again in all studied groups.

### 2.4. Treatment

Four weeks after the beginning of diabetes, the milk thistle and artichoke groups received an oral solution of Livergol® (40 mg/kg) and Atheromod-B® (60 mg/kg), respectively, by gavage once a day for eight weeks. Normal and diabetic groups received an equal amount of distilled water.

The daily doses of drugs for the rats were calculated according to the following formula: dose for rats = (*X* mg/kg ∗ 70 kg ∗ 0.018)/0.2 kg or simplified as 6.3 *X* mg/kg (*X* = the effective dose for man; 70 kg = the standard weight for man; 0.018 = ratio of the equivalent dose for man and rats based on body surface area; 0.2 kg = the standard weight for rats) [25].

### 2.5. Oral Glucose Tolerance and Biochemical Assays

At the end of treatments and after a 12 h fast, a glucose solution (2 g/kg body weight, in 0.9% NaCl) was administered to the rats by oral gavage. FBS was measured 0, 30, 60, 90, and 120 min later using the Glucometer [25].

Erythrocyte superoxide dismutase (SOD) activity and malondialdehyde (MDA) serum levels were determined using the SOD ELISA kit (Randox, UK) and Rat MDA ELISA kit (Cusabio Biotech, China), respectively.

The serum activity of alanine aminotransferase (ALT), aspartate aminotransferase (AST), and alkaline phosphatase (ALP) and the serum levels of total bilirubin (TB), triglyceride (TG), total cholesterol (TC), low-density lipoprotein-cholesterol (LDL-C), and high-density lipoprotein-cholesterol (HDL-C) were measured by routine laboratory methods using an auto-analyzer (Mindray BS-380, China).

### 2.6. Liver Histological Assay

Twelve weeks from the beginning of diabetes, the animals were sacrificed. The euthanasia protocol was carried out by administration of xylazine (10 mg/kg) and ketamine (75–100 mg/kg) to the rats via intraperitoneal injection. The whole liver was resected and fixed with 10% formalin. The liver was sliced serially at1 millimeter thickness, and one slice from the right and left lobes was embedded in tissue capsules. The liver slices were embedded in paraffin, cut into 5 *μ*m sections, and stained with hematoxylin and eosin (H&E). No special staining was done for fat. Samples were observed under a light microscope (Olympus, BX53, Japan) by an expert pathologist in a blind fashion to evaluate the percentage of steatosis. The liver histopathological findings were categorized into three groups: normal, portal tract lymphocytic infiltration, and macrovesicular steatosis.

### 2.7. Statistical Analysis

Data were presented as mean ± SEM. Statistical analysis was determined using one-way analysis of variance (ANOVA) and paired *t*-test. If a significant difference was obtained, the source of difference was located by Tukey's post hoc test. *P* < 0.05 was considered statistically significant. The statistical analysis was performed by the Statistical Package for Social Sciences version 16 (SPSS Inc, Chicago, IL, USA).

## 3. Results

### 3.1. Mortality Rate, Weight Changes, Liver Index, Oral Glucose Tolerance, and Biochemical Assays

At the end of the study, the total mortality rate was 20% (1 in the normal group, 3 in the diabetic group, 2 in the milk thistle group, and 2 in the artichoke group). We measured the weight of rats on the first day (one week after diabetes induction) and the last day (after 12 weeks of diabetic confirmation) and weight changes (the changes between the first day and the last day) and liver index (liver weight divided by body weight) on the last day (Table 1). All groups gained weight significantly, but the comparison among groups revealed that the weight changes were not significant. No difference was seen among groups regarding the liver index (Table 1).

**Figure 1:**
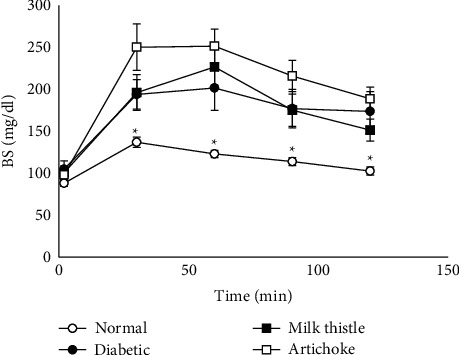
Blood glucose changes in the glucose tolerance test (GTT) in each group. Blood glucose values during the GTT were significantly higher in the diabetic groups (with and without treatment) than in the normal group, which showed insulin resistance in these groups. Values are expressed as the mean ± SEM (*n* = 7–9/group at each time point). Differences were tested by one-way ANOVA followed by Tukey's multiple comparisons test. ^*∗*^*P* < 0.05 indicates a significant difference with the diabetic control group at the same time points.

First-day (one week after the induction) FBS and 28^th^-day FBS of diabetic animals were significantly higher than in normal animals. Blood glucose levels in all three diabetic groups at fasting or 0, 30, 60, 90, and 120 min after glucose administration were significantly higher than those in the normal group (*P* < 0.001). Milk thistle and artichoke had no significant effect on blood glucose level at the first day and 28^th^ day after diabetes induction and on the oral glucose tolerance test, which was performed at the end of the study (about three months after the induction of diabetes) (Figure 1).

The erythrocyte level of SOD in the diabetic group was significantly lower than in the normal group (*P* < 0.001). However, these values for milk thistle- and artichoke-treated groups were significantly higher than those for the diabetic group (*P* < 0.001 and 0.006 for milk thistle and artichoke, respectively). The serum level of MDA in the diabetic group was significantly higher than in the normal group (*P* < 0.001), but the serum levels of MDA for milk thistle- and artichoke-treated groups were significantly lower than those of the diabetic group (*P* < 0.001) after the intervention (Table 2).

Serum levels of different lipid profile components were increased significantly (TG and LDL-C) and nonsignificantly (TC) in the diabetic group compared to the normal group (*P*=0.001, 0.013, and 0.100, respectively). Administration of milk thistle showed a significant decrease in serum levels of TG, TC, and LDL-C (*P* < 0.001, *P*=0.021, and 0.015, respectively). Administration of artichoke showed a significant decrease in the serum level of TG but not TC and LDL-C (*P* < 0.001; Table 3).

In our diabetes model, only the AST level rose significantly and was significantly decreased by milk thistle (*P*=0.039) but not artichoke (Table 4). The ALT, ALP, and TB levels did not change significantly in diabetic animals (Table 4).

### 3.2. Liver Histological Assay

**Figure 2:**
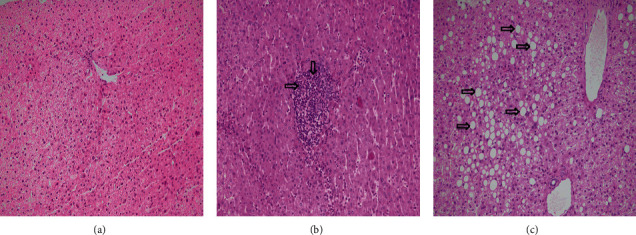
Light microscopic studies of the liver stained with hematoxylin-eosin in each group. (a) Section from the liver tissue shows normal cords of hepatocytes around a central vein. (b) Section from the liver tissue shows moderate lymphocytic infiltration in the portal tract (arrow). (c) Section from the liver tissue shows perivenular macrovesicular steatosis (arrow) (original magnification: 200x).

Histological samples of rats' liver tissue showed normal lobular architecture with central veins and no prominent inflammation, steatosis, or necrosis in the normal group (Figure 2(a)). The hepatic cord structures were changed in the diabetic group, milk thistle-treated group, and artichoke-treated group, and a noticeable accumulation of fat droplets and steatosis could be found (Figure 2(c)). Some lymphocyte infiltration was observed in the diabetic, milk thistle-treated, and artichoke-treated groups (Figure 2(b)).

## 4. Discussion

The present study aimed to assess whether milk thistle (*Silybum marianum* L.) and artichoke (*Cynara scolymus* L.) administration in streptozotocin-nicotinamide-induced diabetic rats would improve glucose tolerance, biochemical markers, hepatic marker parameters, and plasma oxidative stress and also to investigate whether milk thistle and artichoke administration declined the lipid accumulation in the rat liver.

We established an NAFLD rat model of T2DM using STZ + nicotinamide administration. This combination causes partial necrosis of pancreatic *β*-cells. The successful induction of the model was confirmed by basal hyperglycemia at one week and four weeks after injections and the impaired glucose tolerance test at the end of the study (Table 2, Figure 1).

Milk thistle and artichoke had no significant effect on the basal glucose level and oral glucose tolerance test (Table 2, Figure 1). Some observed effects in the current study cannot be attributed to diabetes control (see the following). A similar result with silymarin (an active constituent of milk thistle) has been reported, despite its beneficial effect on the liver and pancreas tissues of diabetic rats [26]. However, there are studies in which these plants or their active substances show hypoglycemic effects. These effects have been proposed to result from preserving or repairing pancreatic beta-cells, decreased insulin resistance, or inhibition of alpha-amylase action [27–30]. The differences in the obtained results may be due to the different doses applied. In our study, the doses were selected based on the recommended dose for hepatoprotection in humans (see methods). However, other causes such as the different experimental models of diabetes and duration of treatment may also have some roles.

In diabetes, lipolysis ensues due to the diminished insulin level and its action. Subsequently, an overload of lipids is deposited in the hepatocytes of animal models, resulting in NAFLD development [31]. In our study, the pathological liver state was categorized as follows (Table 5): *normal* (normal histological structure of the central vein and surrounding hepatocytes); *lymphocyte infiltration* (an increase in liver size and hepatocytes with inflammatory cell infiltration), which is the earliest changes detected in the liver in NAFLD [32]; and *steatosis* (simple hepatic steatosis, also called nonalcoholic fatty liver (NAFL)) that is a relatively benign manifestation of hepatic triglyceride accumulation, with little or no inflammation or liver cell damage [33, 34].

In our study, rat diabetic livers had an obvious accumulation of fat droplets; therefore, steatosis was confirmed (Figure 2(c)).

In this group, AST significantly increased, but no significant changes in ALT, ALP, and TB were observed. In the milk thistle-treated group, fewer and smaller fat droplet accumulation and fewer lymphocytes were observed. In contrast, in the artichoke-treated group, some considerable fat droplet accumulation and steatosis were recognized. In other words, milk thistle showed a mild beneficial effect on the hepatic changes induced by diabetes in the present study and was applied in doses. At the same time, artichoke seemed ineffective in this regard (Table 5).

Several studies have proposed the hepatoprotective effects of silymarin on diabetes [28, 35]. Indeed, histopathological effects and biochemical markers have been more significant than our results in many of them. Again, the differences between the selected doses and the type and experimental model of diabetes may justify these different magnitudes of effects.

There are some reports of hepatoprotective action of artichoke (*Cynara scolymus* L.) in different hepatotoxicity models, but just a few studies on diabetes-induced hepatic changes could be found. Ben Salem et al. reported hepatoprotective effects of the ethanolic extract of leaves of *Cynara scolymus* L. in diabetic rats. They attributed it to the antioxidant and hypolipidemic effects of the plant [30].

Yao et al. demonstrated that administration of silibinin (principal active constituent of silymarin) in rats with NAFLD for six weeks improves fatty degeneration and inflammation in hepatocytes significantly [25]. In the systematic review of Cicero et al., silymarin improved hepatic lipid accumulation after three months of treatment and decreased the 4-year risk of mortality in cirrhosis patients [36]. A randomized clinical trial displayed that silymarin consumption for three months had better results on liver steatosis than lifestyle changes [37]. A randomized controlled trial of biopsy-proven NAFLD patients by Loguercio et al. showed that a daily dose of silibinin induces improvement in histological scores of steatosis, inflammation, and fibrosis [38]. Also, silymarin may reduce liver fibrosis in NAFLD patients [39].

In almost all studies in which the hepatoprotective effects (and other valuable actions) of silymarin and artichoke have been reported, their proper mechanism of action is mainly attributed to their antioxidant effects [27, 28, 30, 35, 40, 41].

These drugs have antioxidant effects through increased SOD (an antioxidant enzyme) levels and decreased MDA (a final lipid-oxidized product) levels on NAFLD in rats. Investigations have shown that artichoke is a valuable source of antioxidants that could have applications in preventing diseases caused by oxidative damage [42]. The use of silymarin can increase the activity of SOD and decrease MDA levels in mice [43] and has protective effects on oxidative stress markers [44, 45].

Interestingly, while just milk thistle showed some hepatoprotective impact in our study, both plant products showed significant antioxidant action, indicated by increased SOD and decreased MDA (Table 2). Based on these results, it can be proposed that the hepatoprotective effect of milk thistle (and silymarin) is not exclusively due to their antioxidant action and other mechanisms seem to be involved. Anti-inflammatory and antifibrotic activities [41] and an increase in irisin [28], a newly discovered polypeptide, are the proposed mechanisms.

Both plant extracts decreased TG significantly in our study, but only milk thistle decreased TC and LDL-C (Table 3). In addition to the above-mentioned biochemical changes, other studies have also reported significant differences in ALT and ALP [46]. However, we did not observe significant changes in these two parameters in all the studied groups. Administration of silibinin in NAFLD rats for four weeks ameliorated TG, TC, and hepatic steatosis significantly [47]. Salomone et al. performed a similar study in a mouse model of fatty liver in which they investigated an in vitro model of hepatocyte lipotoxicity. They used silibinin to treat NAFLD as a modulator of lipid homeostasis. They concluded that silibinin treatment attenuates steatosis, decreases TG and reactive oxygen species (ROS) production, and markedly reduces lipid accumulation in the liver [48]. Also, artichoke attenuates lipid disturbances and has anti-hypercholesterolemic and hepatoprotection effects [19]. It significantly reduced serum lipid profile and the fatty liver deposition in the hepatic lobule in high-fat-diet-induced obese rats [49].

Based on the obtained results, it seems that milk thistle, but not artichoke, might have some hepatoprotective effects in diabetic rats with NAFLD. Its antioxidant action may contribute to this effect, but other mechanisms are possibly involved. Nonetheless, long-term randomized clinical trials will be necessary to understand whether the observed results can be confirmed. Furthermore, the molecular targets and the signaling transduction pathways of plant extracts should still be more deeply investigated.

## 5. Conclusion

Our results showed that milk thistle might have some hepatoprotective effects in diabetic rats with NAFLD. Its antioxidant action may contribute to this effect. Milk thistle also effectively protected the liver from lipid accumulation and severe hepatic changes.

### 5.1. Limitation

One limitation of this study was the short duration to establish and treat the NAFLD rat model. It is better to evaluate the effects of drug treatments in this animal model for a longer time. On the other hand, the strength of our study lies in its histopathologic assessment that could be considered a basis for future clinical trials.

## Figures and Tables

**Table 1:** Weight on the first day and last day, body weight changes, and liver index values.

Group	Weight (g)		Body weight changes (g)	Liver index
	First day	Last day		
Normal	240.2 ± 1.2	311.1 ± 7.6^‡^	70.91 ± 7.07	2.7 ± 0.1
Diabetic	240.1 ± 1.8	305.4 ± 8.8^‡^	65.3 ± 8.05	2.9 ± 0.2
Milk thistle	240.3 ± 1.9	314 ± 13^‡^	73.7 ± 12.15	2.8 ± 0.1
Artichoke	238.8 ± 2	280.5 ± 14.5^†^	41.7 ± 13.6	3 ± 0.2

Values are expressed as the mean ± SEM (*n* = 7–9/group). Weight of the first day, the weight of the last day, body weight changes, and liver index. Differences between the weights of the first day and the last day were tested by the paired *t* test, and ANOVA was used to test the differences of the bodyweight changes and liver index between groups. †*P* < 0.05 versus first day; ‡*P* < 0.001 versus first day.

**Table 2:** Biochemical marker values.

Group	FBS (mg/dl)		SOD (unit/ml)	MDA (*μ*m/l)
	Day-7	Day-28		
Normal	92 ± 2^‡^	94 ± 2^‡^	259.6 ± 23.2^‡^	0.6 ± 0.0^‡^
Diabetic	173 ± 9	178 ± 10	88.1 ± 14.6	1.7 ± 0.2
Milk thistle	151 ± 8^†^	165 ± 10	206.2 ± 17.6^‡^	1.0 ± 0.1^‡^
Artichoke	156 ± 5	167 ± 7	171.7 ± 18.1^†^	1.2 ± 0.9^‡^

Values are expressed as the mean ± SEM (*n* = 7–9/group). FBS, fasting blood sugar; SOD, superoxide dismutase; MDA, malondialdehyde. Differences were tested by one-way ANOVA and Tukey's post hoc test. †*P* < 0.05 versus the diabetic group; ‡*P* < 0.001 versus the diabetic group.

**Table 3:** Lipid profile component values.

Group	TG (mg/dl)	TC (mg/dl)	LDL-C (mg/dl)
Normal	14.9 ± 1.7^‡^	45.8 ± 2.3	10.5 ± 1.0^†^
Diabetic	45.0 ± 1.4	59.1 ± 6.5	16.7 ± 1.5
Milk thistle	21.6 ± 1.1^‡^	39.4 ± 7.3^†^	10.2 ± 1.8^†^
Artichoke	23.2 ± 2.9^‡^	52.0 ± 5.3	13.5 ± 2.2

Values are expressed as the mean ± SEM (*n* = 7–9/group). TG, triglyceride; TC, total cholesterol; LDL-C, low-density lipoprotein-cholesterol. Differences were tested by one-way ANOVA and Tukey's post hoc test. †*P* < 0.05 versus the diabetic group; ‡*P* < 0.001 versus the diabetic group.

**Table 4:** Hepatic marker parameter values.

Group	ALT (U/L)	AST (U/L)	ALP (U/L)	TB (mg/dl)
Normal	91.1 ± 10.3	138.1 ± 7.3^‡^	105.9 ± 14.3	3.0 ± 0.7
Diabetic	95.0 ± 20.8	254.3 ± 15.9	110.4 ± 16.0	2.0 ± 0.8
Milk thistle	71.2 ± 8.6	193.4 ± 23.8^†^	239.4 ± 88.7	2.9 ± 0.5
Artichoke	62.5 ± 8.3	306.9 ± 25.4	242.0 ± 83.0	2.3 ± 0.6

Values are expressed as the mean ± SEM (*n* = 7–9/group). ALT, alanine aminotransferase; AST, aspartate aminotransferase; ALP, alkaline phosphatase; TB, total bilirubin. Differences were tested by one-way ANOVA and Tukey's post hoc test. †*P* < 0.05 versus the diabetic group; ‡*P* < 0.001 versus the diabetic group.

**Table 5:** Liver histopathological changes.

Group	Pathology state			Total
	Normal *N* (%)	LI *N* (%)	Steatosis *N* (%)	
Normal	9 (100.0)	0 (0.0)	0 (0.0)	9 (100.0)
Diabetic	0 (0.0)	2 (28.6)	5 (71.4)	7 (100.0)
Milk thistle	0 (0.0)	6 (75.0)	2 (25.0)	8 (100.0)
Artichoke	0 (0.0)	2 (25.0)	6 (75.0)	8 (100.0)
Total	9 (28.1)	10 (31.3)	13 (41.6)	32 (100.0)

The values show the frequency of liver pathological state (normal, lymphocyte infiltration (LI), and steatosis) in each group.

## Data Availability

All data are included within the manuscript.
